# Autoimmune Haemolytic Anaemia as a Rare and Potentially Serious Complication of Crohn’s Disease in a 11-Year-Old Child—Case Report and Minireview

**DOI:** 10.3390/children10101698

**Published:** 2023-10-17

**Authors:** Aleksandra Dybowska, Aneta Krogulska

**Affiliations:** Department of Paediatrics, Allergology and Gastroenterology, Ludwik Rydygier Collegium Medicum in Bydgoszcz, Nicolaus Copernicus University, 85-067 Torun, Poland

**Keywords:** Crohn’s disease, autoimmunological haemolytic anaemia, children

## Abstract

Inflammatory bowel disease (IBD) is the term given to a heterogeneous group of chronic inflammatory diseases of the gastrointestinal tract (GI). These include ulcerative colitis (UC), where the inflammatory process involves only the intestinal mucosa, and Crohn’s disease (CD), where it can involve the entire wall of the GI in all of its sections. In addition to typical gastrointestinal complaints, IBD manifests with a range of extraintestinal symptoms involving inter alia the eyes, joints, skin, liver and biliary tract. These can cause a number of extraintestinal complications; of these, one of the most common is anaemia, usually resulting from nutritional deficiencies, especially iron, or chronic inflammation. When treating patients with IBD, it is important to consider the possibility of rare but serious complications, including autoimmune haemolytic anaemia (AIHA). This condition occurs in only 0.2 to 1.7% of UC cases and is even rarer in CD. AIHA is usually mild but can occur suddenly and cause very rapid anaemia. In the article presented here, we describe the case of a patient who developed AIHA two years after a diagnosis of CD, causing a life-threatening diagnostic and therapeutic challenge for the medical team.

## 1. Introduction

Inflammatory bowel diseases (IBDs) are characterized by chronic inflammation of the intestinal wall and a chronic and recurrent course. The group includes ulcerative colitis (UC) and Crohn’s disease (CD) [1,2,3]. The pathogenesis of IBD is not yet fully understood, but its development is known to be influenced by the dysregulation of specific and non-specific immune responses, environmental factors, gut microbiota and genetic factors [3,4]. Although IBD is a disease of the gastrointestinal tract, its symptoms are not limited to this system, and it may co-occur with arthropathy, erythema nodosum and primary sclerosing cholangitis (PSC) [4,5]. Various types of extraintestinal complications can also be observed, both as a result of ongoing inflammation and as an adverse effect of the treatments [2]. Up to 50% of IBD patients experience at least one extraintestinal complication, of which up to 24% are detected before the diagnosis of IBD [2,3], and 29% occur for as long as 15 years following the disease [5].

One of the most common complications of IBD is anaemia [1,2,4]. It is observed in 6–74% of patients with IBD, depending on the definition of anaemia and the patient group considered [2]. The most common forms of anaemia observed in IBD are the iron deficiency form, which can result from gastrointestinal bleeding or malabsorption, or chronic disease anaemia, which results from the suppression of erythropoiesis or reduced iron absorption [1,2,4,6]. Other common causes of anaemia are vitamin B12 or B9 deficiency and the side effects of IBD medications (e.g., sulfasalazine and methotrexate) [1,2,4].

Autoimmune haemolytic anaemia (AIHA) is a heterogeneous group of cytopenia conditions caused by the lysis of erythrocytes due to antibodies coating the cells [7,8,9]. AIHA may be a primary disease in 50% of cases and secondary in the remaining 50%, associated with inter alia autoimmune diseases, immunodeficiencies, infections, transplants, lymphoproliferative diseases or drugs [2,3,4,7,8,9,10]. Based on the thermal characteristics of the antibodies and the Coombs test, AIHA can be further subdivided into two types: the more common warm IgG antibody-associated anaemia (wAIHA—warm autoimmune haemolytic anaemia) noted in 80% of cases, and the less common cold IgM antibody-associated anaemia (CAD—cold agglutinin disease) [3,7,8]. These antibodies coat red blood cells, thus making them targets for macrophages in the liver and spleen; they are then phagocytosed, resulting in anaemia [11,12].

AIHA is a rare complication of IBD; it can accompany 0.2–1.7% of UC cases and even fewer CD cases [1,2,6,10,13]. Toplicanin et al. reported that only seven cases of AIHA in patients with CD had been documented by the year 2020, including one in an 11-year-old child, and described an eighth case involving a 36-year-old woman [2]. Since then, two more adult patients have been described [14,15]. Therefore, the patient described herein is only the eleventh case published to date. A summary of all published cases of AIHA in the course of IBD to date is shown in Table 1.

## 2. Case Description

The present study described the case of a girl diagnosed with CD at the age of nine years, who later developed a severe and rare complication of autoimmune haemolytic anaemia at the age of 11.

The patient was first hospitalized aged nine years in an infectious disease hospital for a fever (up to 39.5 C) that persisted for 1.5 months. Due to the COVID-19 pandemic, she was first suspected of having SARS-CoV-2 infection. This was ruled out, and the girl was transferred to the paediatric ward. The patient presented no other symptoms; however, her parents reported that the child had a low bodyweight (weight 20 kg, <3 centiles, height 133.5 cm, 25–50 centiles, BMI 11.22, <3 centiles). The family history was unencumbered. The concentration of faecal calprotectin was 1500 mg/g. A colonoscopy identified inflammatory lesions, multiple ulcers and polyps in the ileocecal region, and from the mid-ascending colon to the mid-transverse colon. CD was suspected, but histopathology described non-specific inflammatory infiltrates. To verify the diagnosis, an abdominal CT scan and magnetic resonance imaging (MRI) with enterography were additionally performed; the results confirmed lesions typical of IBD, thus confirming the diagnosis of CD. The onset of the disease, which was manifested only by a fever that lasted for six weeks, was described in more detail in a previous article [19]. The present article presents a rare and dangerous complication of Crohn’s disease, AIHA.

After the diagnosis of CD, remission was induced by systemic steroid therapy. Taking into consideration the patient’s poor general condition, it was decided that she would benefit more from systemic steroid therapy rather than from EEN (Exclusive Enteral Nutrition). However, the condition demonstrated an unsatisfactory response to treatment from its commencement; initially, a lack of response to systemic steroids was noted, and then antibodies developed after adalimumab administration. During this time, a second endoscopic examination was performed. The colonoscopy identified active changes in the ileocecal region and from the caecum to the hepatic flexure with deep ulcers and polyps. Histopathology described once again non-specific inflammatory infiltrates. This endoscopic funding confirmed the deteriorating answer to applied treatment. Although infliximab was eventually used (5 months after CD diagnosis), with improvements in disease control, the girl’s disease control deteriorated some months later, at the age of 11, when she was hospitalized twice for exacerbation of *Clostridium difficile* (*C. difficile*) infection. Due to the recurrence of the infection, oral vancomycin treatment was administered, with improvement in the patient’s clinical condition.

After one month of vancomycin therapy, the patient again presented to the hospital following an exacerbation of her disease. During the hospitalization, generalized oedema and transudates into body cavities were observed; these increased over time, despite compensating hypoalbuminemia. An abdominal CT scan was performed, ruling out GI perforation and bleeding. Cardiac ECHO ruled out myocardial contractile dysfunction. Laboratory tests showed high CRP levels with negative PCT, reduced protein and albumin levels, slight hypokalaemia, high NTpro-BNP levels and worsening normocytic anaemia. Taking into account the whole clinical picture and laboratory results, a test for antibodies to SARS-CoV-2 was performed, ruling out coronavirus disease (COVID-19). Criteria for paediatric inflammatory multisystem syndrome associated with coronavirus disease (PIMS) was analysed. Our patient did not fulfil WHO criteria for PIMS that was used at the time of hospitalization. Empirical antibiotic therapy with ceftriaxone and metronidazole was started and oral vancomycin therapy continued. Furosemide was included in the treatment, achieving a reduction in oedema and weight loss.

**Figure 1:**
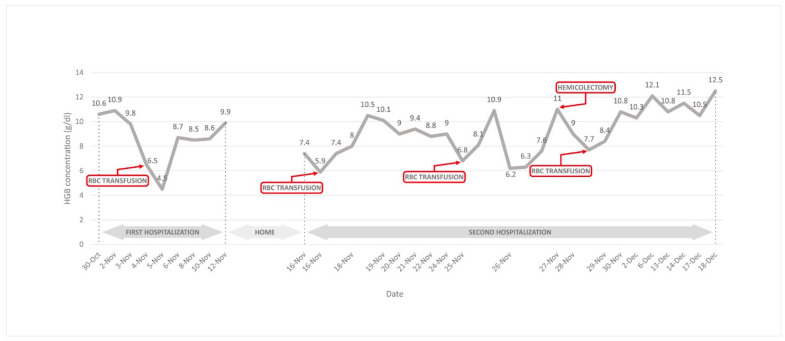
Changes in haemoglobin concentration during first and second AIHA episode.

Due to the rapidly decreasing haemoglobin (HGB) concentration, from 9.8 g/dL to 6.5 g/dL, the girl required a transfusion of irradiated leukocyte-depleted red blood cell concentrate (Figure 1). However, about 40 min after the transfusion (group compatible), the patient began vomiting while drinking an endoscopy preparation and passed out. Another brief loss of consciousness occurred after passing a stool. In an urgent morphology test, the HGB concentration could not initially be determined, and the laboratory reported the possibility of cold antibodies. On retesting, the HGB level was found to be 4.5 g/dL. As no expected increase in HGB concentration was observed after the blood transfusion, in the absence of signs of bleeding, it was proposed that the observed anaemia may have had a haemolytic basis. Acute haemolytic crisis in the course of intestinal infection was diagnosed.

The girl was consulted haematologically and AIHA was suspected. Immediately, methylprednisolone was administered. Serological examination yielded a positive Coombs test in the blood sample, with red blood cells coated with IgG-class antibodies, and the serum showed the presence of cold agglutinins, which confirmed the diagnosis of AIHA. Treatment with steroids was sustained and an irradiated leukocyte-depleted red blood cell concentrate was transfused again, this time achieving an increase in HGB and a significant improvement in the girl’s general condition. As steroid therapy continued, a further increase in HGB levels was noted, together with a rise in reticulocytes, indicating normal marrow recovery from haemolytic crisis. At discharge, the girl was recommended vancomycin treatment in tapering doses to prevent another *C. difficile* infection. 

Almost four weeks later, the patient was hospitalized again for another exacerbation of the disease. On admission, laboratory tests again showed anaemia (HGB 7.4 g/dL), with reticulocytosis and elevated levels of HGB free (88 mg/100 mL). An irradiated leukocyte-depleted red blood cell concentrate was transfused once, resulting in a stable but still low HGB concentration (7.4 g/dL). Microbiological examination of the stool ruled out a recurrence of *C. difficile*. Markers of hepatotropic virus infection were negative (hepatitis B surface antigen and the hepatitis C antibody test). It was decided to perform an MRI with enterography, which showed active lesions of the terminal ileum and dilatation of the large intestine. Due to the patient’s poor general condition, together with MRI enterography findings, total parenteral nutrition was started. Another factor that contributed to making this decision was the girl’s lack of appetite and weight loss. Subsequently, cyclosporine (at a dose of 5 mg/kg/day i.v.) was added to the treatment and broad-spectrum antibiotic therapy (ceftazidime, metronidazole and amikacin) was included. Although a temporary improvement in her clinical condition was achieved, after a few days, the patient developed fluctuations in heart rate, from bradycardia (54/min) to tachycardia (120/min), accompanied by renewed anaemia (6.8 g/dL) with reticulocytosis. Transfused irradiated leukocyte-depleted red blood cell concentrate achieved an increase in HGB concentration, accompanied by rapid repeated anaemia (about 4 g% within an hour) with reticulocytosis. The dynamics of the changes in HGB concentration are shown in Figure 1. 

Again, blood was transfused. During this period, the girl began to develop facial swelling and her abdominal circumference increased. Despite the inclusion of salvage therapy with cyclosporine, due to active gastrointestinal bleeding, the patient was qualified for a right-sided hemicolectomy. After surgery, parenteral nutrition and antibiotic therapy (imipenem and vancomycin) were administered. Two days after the procedure, the patient required a transfusion of an irradiated leukocyte-depleted red blood cell concentrate due to anaemia; this time, the anaemia resulted from blood loss during the procedure as no products of haemolysis were noted.

The postoperative period was complicated by posterior reversible encephalopathy syndrome (PRES). Treatment was modified accordingly, discontinuing cyclosporine and including an antiepileptic drug, resulting in the neurological condition improving. After the surgery, the gastrointestinal complaints were found to resolve, improvements were noted in haematological parameters and the ultrasound imaging of the intestines, and the inflammatory markers were seen to normalize. Systemic steroid therapy was administered in decreasing doses, with no haemolysis observed. Azathioprine was included as a follow-up treatment for exacerbations of the underlying disease. Just before the scheduled discharge, the patient was diagnosed with another *C. difficile* infection. Vancomycin was included, with clinical improvement.

Table 2 presents results of complete blood tests and changes in parameters during the first and second episode of AIHA. It can be noticed that at the time of first AIHA episode, the patient presented with microcytic hypochromic anaemia, which could have suggested iron deficiency form. However, taking into consideration the lack of improvement after transfusion, together with results of additional laboratory tests (elevated total bilirubin and suspected presence of cold antibodies), haemolytic basis of anaemia was suspected. It was confirmed when a gradual improvement in blood parameters with reticulocytosis was noted after steroid treatment. Elevated platelet levels during this time can be a result of acute blood loss. At the admission during the second hospitalization, when AIHA was diagnosed, the patient presented with normocytic normochromic anaemia. Having in mind the previous episode of AIHA, haemolytic parameters were marked and elevated levels of HGB free and total bilirubin together with reticulocytosis once more indicated that it was haemolytic anaemia. However, it did not respond well to transfusion, and this time, the patient eventually required a hemicolectomy. After the operation, normocytic normochromic anaemia once more was diagnosed. At that time, haemolytic parameters were not elevated which could indicate that the cause of anaemia was blood loss after the operation. After a transfusion, HGB initially increased. Unfortunately, at the end of hospitalization, the patient was diagnosed with C. difficile infection which could cause periodic decreasing of HGB levels. A month later, the patient was readmitted for a third recurrence of *C. difficile*. Oral treatment with vancomycin was started, with a gradual improvement seen in the clinical condition. Due to transient anaemia with features of haemolysis, the planned reduction in the steroid treatment was halted to prevent the recurrence of autoimmune haemolytic anaemia. Following the recurrence of *C. difficile* infection, it was decided to transplant intestinal microbiota to the duodenum.

Currently, the patient remains under the care of the gastroenterology clinic. Azathioprine is used as a prevention of relapses. At the last follow-up visit, the disease activity scored 0 on the PCDAI scale. This was confirmed in the last endoscopic examination, when, in the colonoscopy, considerable regression of inflammation was described. To this day, the patient remains in remission.

## 3. Discussion

Like Crohn’s disease (CD), AIHA is an autoimmune disease, and it is known that autoimmune diseases tend to co-occur [4,10]; however, a diagnosis of AIHA can precede that of IBD, coincide with it, or occur up to several years after a colectomy [2,6,13]. Although the exact pathomechanism of AIHA in CD is unknown, it has been proposed that following damage caused by inflammation, the intestinal wall absorbs non-specific antigens, not erythrocyte antigens, which stimulate an immune response. Following a breach in the intestinal wall, autoantibodies are produced which react with erythrocyte membrane antigens [2,3,10,17,20].

The described mechanism indicates that the course of AIHA may be related to the activity and severity of IBD [1,2,11]. This is all the more likely when AIHA appears as the first symptom of IBD or during its course. This hypothesis is also supported by previous data indicating that AIHA was only cured by achieving IBD remission through surgical removal of the inflamed bowel [2,11,17,20,21,22]. In addition, the fact that AIHA can be cured in patients with IBD by performing a colectomy, i.e., without needing a splenectomy, also suggests that it is the inflammation-altered intestinal wall that is responsible for autoantibody production [6]. All previously described patients with AIHA were in the active phase of the disease at the time of diagnosis [2].

In the case of our patient, the diagnosis of AIHA also correlated with CD exacerbation. However, as the endoscopic examination was not performed due to the patient passing out during preparation, it is unknown how extensive the intestinal lesions were at the time. Moreover, during the re-exacerbation of the underlying disease, with active lesions present in the terminal ileum and dilation of the large intestine, the girl again became anaemic, with products of haemolysis, which could not be controlled by transfusing blood products. Only surgical removal of a section of the inflamed intestine helped slow haemolysis.

Another possible pathomechanism for the development of AIHA in IBD patients is infection, which can induce red blood cell haemolysis through various mechanisms, such as modification of erythrocyte membrane antigens, activation of polyclonal B cells or molecular mimicry [8,9,23]. In the event of AIHA occurring in children, and with the co-occurrence of cold agglutinins [9,12], as in our present patient, it is likely that infections may be a causative factor. AIHA has most commonly been associated with parvovirus and hepatotropic virus infection, and in the case of bacteria, with *Mycoplasma pneumoniae*, *Mycobacterium tuberculosis* and *Brucellosis* infection [9].

As the diagnosis of AIHA and its recurrence in the child described herein coincided with *C. difficile* infections, it was also supposed that the autoimmune process underlying the haemolysis may be triggered by recurrent infection. However, no reports indicating that AIHA may be caused by *C. difficile* infection could be identified in the literature. Hence, it is difficult to decisively confirm whether *C. difficile* was the cause of AIHA or whether the IBD exacerbation itself triggered this complication. Anti-inflammatory signalling triggered by a bacterial infection can initiate a cycle of inflammation, increasing the permeability of the intestinal wall and facilitating its penetration, which then, as mentioned above, can activate autoantibody production, thus potentially leading to the development of AIHA [24].

On the other hand, it is also possible that the infection itself is not the cause of AIHA, but that it may arise as a complication of treatment [9]. The immunomodulation associated with the pathogenesis of IBD, and the immunosuppression used in its treatment, can raise the risk of infection. Indeed, AIHA treatment commonly employs immunosuppressive drugs which disrupt immunity. These overlapping factors disrupt the immune system, further increasing the risk of infection in IBD patients by up to 30% [9,15].

The fact that AIHA can arise as a complication following the treatment of IBD alone is significant. To date, such cases have been documented in patients treated with sulfasalazine [25,26], vedolizumab [11] and infliximab [14,27,28,29,30]. Vermiere et al. studied cases of autoimmunity in 125 CD patients during a one-year follow-up after infliximab therapy; the findings indicate a 0.8% incidence of AIHA as a complication of infliximab treatment [27,28]. Given this possibility, it is important to consider at what point AIHA appeared. If it first occurred even before the drug was introduced, this mechanism is unlikely. However, in the case of our patient, AIHA was diagnosed during infliximab therapy; as such, it seems possible that her development of AIHA may have been related to the therapy.

In most cases, AIHA has a benign and easily treatable course. However, it sometimes has an acute onset and requires urgent intervention [7,8,12]. Multiple recurrences are also possible, making it a chronic problem [7]. The course of wAIHA is usually milder than CAD [1]. While AIHA remission can be achieved by treating the underlying disease and reducing its activity [1,6,10], this is sometimes not possible due to the rapid onset of the disease; in such cases, it is necessary to use pharmacotherapy. The first choice for treating AIHA in patients with IBD is corticosteroid treatment. However, positive results have been reported for treatment with other immunosuppressants (rituximab, mycophenolate mofetil, cyclophosphamide and azathioprine) [1,2,9,12,15]. In such situations, physicians are faced with a choice: whether to risk accumulating immunosuppressive drugs in a patient with IBD, or to discontinue IBD medications for the duration of AIHA therapy and risk exacerbating the underlying disease [15].

AIHA, especially recurrent AIHA, can also be cured by splenectomy, but such treatment is not recommended in patients with IBD; it is worth trying immunosuppressive drug therapy before making such a radical decision [6,10]. In addition, it should be remembered that the effectiveness of a splenectomy depends on the type of AIHA. A splenectomy may be an effective treatment in the case of wAIHA, where haemolysis takes place extravascularly, mainly in the spleen [7]. In contrast, in CAD, haemolysis generally occurs intravascularly in the liver, and a splenectomy would not be suitable; however, in the most severe cases of CAD, haemolysis can also take place in the spleen [1,7,8].

Despite pharmacological treatment, in some cases only surgical intervention and resection of a section of the intestine can treat AIHA [3,11,17,20,21,22], as was the case for our patient. Despite chronic corticosteroid therapy, AIHA re-emerged as another exacerbation, causing rapid anaemia and contributing to the decision to perform a hemicolectomy.

Analysing previous cases (Table 1), it can be concluded that AIHA in the course of CD is more common in men than in women, with a mean age at diagnosis of 34.09 years. Interestingly, it appears that only one case involving an 11-year-old boy has been described so far; as such, our patient, also 11 years old, is the second described minor diagnosed with AIHA in the course of CD. In most cases, as in our patient, AIHA developed after the diagnosis of CD. Two cases also considered the influence of the drug as a causative factor in the pathogenesis of AIHA [14,15].

Regarding the present diagnosis, taking into account the clinical picture, previous documented immunization (production of antibodies to adalimumab) and the opinion of the consulting experts (gastroenterologist, haematologist, immunologist and surgeon), it seems that autoimmune haemolytic crisis could have manifested as a complication of the applied biological treatment. However, it cannot be excluded that the presence of a recurrent *C. difficile* infection may have been involved in the pathogenesis of AIHA, especially since during its third recurrence, the patient was again diagnosed with anaemia due to haemolysis, despite the fact that the pathologically altered bowel fragment had been previously removed.

It is essential to mention that even though the patient is no longer under infliximab treatment, she still requires close monitoring for possible side effects of medication used in the supportive therapy. Our patient, up to now, is treated with azathioprine, of which side effects can include, e.g., pancreatitis, leucopenia, gastrointestinal intolerance, myelotoxicity, hepatotoxicity and infectious complications [31,32].

## 4. Conclusions

The symptoms arising in the course of IBD can affect not only the GI tract, but also many other systems. A common complication is anaemia. AIHA is a rare complication but one that is potentially life-threatening. The description presented here is the second published case of AIHA in a child with CD. An awareness of such complications is essential in the management of children with IBD. Taking into consideration this rare complication, it is possible to mark the levels of haemolysis products in the early stages of acute anaemia in patients with CD. This can lead to quicker diagnosis and treatment of AIHA.

## Figures and Tables

**Table 1:** Characteristics of previously reported cases of CD patients with AIHA according to Toplicanin et al. [2] in own modification.

	Gender	Age (Years)	Disease That Occurred First	Time between Diagnosis of CD and AIHA	Scope of CD Changes	Coombs Test	Therapy That Did Not Induce Remission of AIHA	Therapy That Induced Remission of AIHA	**CD Treatment at the Time of AIHA Diagnosis**	**Comorbidities**
Eliam, 1993 [16]	M	41	AIHA	7 years	Colitis (cecum and ascending colon)	Positive	CS	Splenectomy	-	Primary sclerosing cholangitis
Hochman, 2002 [6]	M	11	CD	2 years	Ileocolitis	Positive	CS Mesalamine 6-MP	MTX	Mesalamine	-
Ng, 2004 [10]	M	44	CD	2 years	Ileitis with perianal abscess and fistula	Positive	Mesalamine	CS AZA	CS	-
Plikat, 2005 [17]	M	29	Simultaneously	0	Ileocolitis with proximal disease (duodenum)	Positive	CS MTX Cyclosporine anti-TNF	Subtotal colectomy	-	-
Tsiopulos, 2009 [13]	M	57	AIHA	*	Ileocolitis	Positive	*	CS	-	Thrombosis of ophthalmic artery
Kallel, 2009 [18]	M	21	CD	9 months	Ileocolitis with Perianal fistula	Positive	Mesalamine	CS	Mesalamine	Primary sclerosing cholangitis
Park, 2014 [4]	F	41	CD	4 years	Ileitis	Negative	*	CS	Mesalamine	-
Al.-Ansari, 2021 [14]	M	63	CD	*	*	Negative	*	CS	Infliximab (second induction dose)	Hypertension
Cheah, 2021 [15]	M	21	CD	*	Left-sided disease	Positive	CS	rituximab	vedolizumab (first maintenance dose)	-
Toplicanin, 2022 [2]	F	36	AIHA	3 months	Colitis	Positive	CS anti-TNF AZA	vedolizumab	-	-
Our patient	F	11	CD	2 years	Ileocecal region and from the mid-ascending colon to the mid-transverse colon	Negative	CS Cyclosporine	Right-sided colectomy	Infliximab (fourth maintenance dose)	-

M—male, F—female, AZA—azathioprine, CS—corticosteroids, 6-MP—6-mercaptopurine, MTX—methotrexate, * not reported.

**Table 2:** Changes in complete blood count during first and second AIHA episode.

	Day of Hospitalization	WBC (4.50–13.50 K/mcL)	RBC (3.80–5.00 Million Cells/mcL)	HGB (12.0–15.0 g/dL)	HCT (34.0–43.0%)	MCV (73.0–95.0 fl)	MCH (25.0–32.0 pg)	MCHC (32.0–37.0 g/dL)	PLT (175–345 K/mcL)
First AIHA episode	1	8.38	4.96	10.6 ↓	34.0	68.5 ↓	21.4 ↓	31.2 ↓	533 ↑
	3	9.36	5.03 ↑	10.9 ↓	36.2	72.0 ↓	21.7 ↓	30.1 ↓	447 ↑
	4	7.2	4.42	9.8 ↓	31.3 ↓	70.8 ↓	22.2 ↓	31.3 ↓	418 ↑
	5	9.16	2.86 ↓	6.5 ↓	21.4 ↓	74.8	23.8 ↓	31.8 ↓	462 ↑
	RBC transfusion								
	6	23.35 ↑	1.75 ↓	4.5 ↓	13.6 ↓	77.7	25.7	33.1	253
	8	22.89 ↑	3.51 ↓	8.7 ↓	23.8 ↓	67.8 ↓	24.8 ↓	36.6	498 ↑
	9	25.01 ↑	3.44 ↓	8.5 ↓	23.6 ↓	68.6 ↓	24.7 ↓	36.0	558 ↑
	11	32.73 ↑	3.43 ↓	8.6 ↓	24.3 ↓	70.8 ↓	25.1	35.4	570 ↑
	13	34.96 ↑	3.86	9.9 ↓	28.1 ↓	72.8 ↓	25.6	35.2	657 ↑
Second AIHA episode	1	9.24	3.42 ↓	9.1 ↓	26.1 ↓	76.3	26.6	34.9	411 ↑
	2	7.23	2.28 ↓	5.9 ↓	17.4 ↓	73.6	25.9	33.9	300
	RBC transfusion								
	3	4.87	3.91	10.5 ↓	31.1 ↓	79.5	26.9	33.8	353 ↑
	5	6.53	3.27 ↓	9.0 ↓	25.6 ↓	78.3	27.5	35.2	340
	8	8.27	3.00 ↓	8.2 ↓	24.9 ↓	83.0	27.3	32.9	334
	10	9.89	2.49 ↓	6.8 ↓	20.5 ↓	82.3	27.3	33.2	237
	RBC transfusion								
	10	13.01	3.80	10.9 ↓	31.5 ↓	82.9	28.7	34.6	254
	11	8.09	2.21 ↓	6.2 ↓	18.0 ↓	81.4	28.1	34.4	195
	12	9.24	3.88	11.1 ↓	31.7 ↓	81.7	28.6	35.0	215
	Hemicolectomy								
	13	14.94	3.12 ↓	9.0 ↓	26.1 ↓	83.7	28.8	34.5	182
	13	9.89	2.68 ↓	7.7 ↓	22.5 ↓	84.0	28.7	34.2	187
	RBC transfusion								
	15	12.32	3.81	10.8 ↓	31.6 ↓	82.9	28.3	34.2	243
	21	13.88 ↑	4.08	12.1	35.8	87.7	29.7	33.8	377 ↑
	28	14.51 ↑	3.68 ↓	10.8 ↓	31.7 ↓	86.1	29.3	34.1	372 ↑
	35	9.38	4.34	12.5	38.4	88.5	28.8	32.6	300

WBC—white blood cells, RBC—red blood cells, HGB—haemoglobin, HCT—haematocrit, PLT—platelet, MCV—mean corpuscular volume, MCH—mean corpuscular haemoglobin, MCHC—mean corpuscular haemoglobin concentration, ↑—elevated level, ↓—decreased level.

## Data Availability

Data sharing not applicable.
